# Supplementary material for: Integrating human papillomavirus testing as a point-of care service using GeneXpert platforms: Findings and lessons from a Kenyan pilot study (2019–2020)
Source: PLoS One. 2023 May 25;18(5):e0286202. doi: 10.1371/journal.pone.0286202 (PMC10212151; doi:10.1371/journal.pone.0286202)
Supplement: Attachment — Submitted filename: Plosone REVIWER integrating Human.docx [file pone.0286202.s001.docx]

**Integrating human papilloma virus testing as a point-of care service using GeneXpert platforms: 2 Findings and lessons from a Kenyan pilot study (2019-2020)**

**Abstract**

**Key Findings:**

**line 61:** is 52.8% instead 53.8% please review

**Data management**

Page 15: lines 201 and 202: table is not referenced in text.

**Findings**

**Screening performance indicators
Page 16:**

Line 231: Table 1: HPV testing statistics, National HPV testing pilot project, Kenya, 2020: is not referenced in text.

**Gene Xpert utilization**

**Page 19:** Table 4: Change in utilization of gene Xpert equipment during the HPV pilot, Kenya, 2020: is not referenced in text.

**Page 19:** **Successes, challenges and opportunities**

Table 5: Successes, challenges and opportunities identified during the nominal group discussions, Kenya HPV pilot study, 2020: is not referenced in text.

**Line 277: is pandemic? Or panemic?**

**Discussion**

- Page 23, line 364 is implementation

**Conclusion and recommendations**

**Page 24 line 376:** …. After future only a dot.
