# Supplementary material for: Integrating human papillomavirus testing as a point-of care service using GeneXpert platforms: Findings and lessons from a Kenyan pilot study (2019–2020)
Source: PLoS One. 2023 May 25;18(5):e0286202. doi: 10.1371/journal.pone.0286202 (PMC10212151; doi:10.1371/journal.pone.0286202)
Supplement: Attachment — Submitted filename: Response to reviewers_HPV pilot_19March23.docx [file pone.0286202.s002.docx]

March 11, 2023

The Editor

PLOS journal

RE: Response to reviewer’s comments for the manuscript; **Integrating human papillomavirus testing as a point-of care service using GeneXpert platforms: Findings and lessons from a Kenyan pilot study (2019-2020)**

Thank you for getting our manuscript through peer review. We have noted with gratitude the kind comments from the two reviewers. We have also made efforts to incorporate the reviewers’ advice as much as possible. Below, we present both the comments from the reviewers, as well as our responses (in italics), and link to the section of the manuscript. In addition, we have provided the revised manuscript, both with the revised sections highlighted in yellow, as well as a clean copy.

**First reviewer**

**Comment 1**: In line 143, authors mentioned that population was calculated from the 2019 Kenya census. I suggest that the authors may include total number of women in this section.

***Our response****: We have added the number of women 30-49 years in the six counties, which is 16,666 (line 143).*

**Comment 2**: Pilot implementation approach: Authors mentioned that the pilot had three phases. I think the authors should include some further detail regarding these phases. This information could improve the methods section. For example, it could be useful a deeper description about training (e.g., which content was included in training sessions? How many training sessions were carried out?); community-based self-sampling approach (Who offered the Self-collection? Where they offered it? Did they use any communication material to explain how to do self-collection?); Delivery of results (Who explain HPV-results and the following steps? The personnel in charge of delivery of HPV- results had received training about it?
Regarding evaluation phase more details regarding NGT exercises are also needed (for example framework, methodology, etc.). In Analysis section they could include details regarding qualitative analysis of data from the NGT exercises.

***Our response****: this has now been addressed in detail (see lines 152-157; 164-168; 195-200; and 226-227).*

**Comment 3: Results**
Screening and follow up indicators: It could be useful, if it is possible, that authors report screening and follow up percentages among women who perform clinician collected and self-collected samples. Adherence to follow up could be lower among women with self-collected samples than among women who attend to health centers to be screened.

***Our response****: Unfortunately, we did not segregate the follow-up data on the basis of sample collection technique. However, we carried out a second pilot, assessing feasibility of a sample-referral strategy to national laboratories, which included that segregation. Those findings are being written-up.*

**Comment 4: Discussion**
Authors pointed out that “only 10.0% had evidence of VIA being done”. They mentioned that a possible explanation may be that more women underwent VIA, but documentation in the screening registers was incomplete, especially due to the fact that VIA was performed on a different visit from the initial screening visit”. Maybe, if the VIA was performed on a different visit from the initial screening, it could be possible that other institutional, subjective or economic barriers could be explain the low adherence rate. Evidence regarding follow up adherence showed that women in low-middle resource setting face a lot of institutional, cultural or economic barriers that reduce adherence to follow up. I suggest that authors could discuss some of these barriers or explain better why the considerer that it is due of lack of registration.

***Our response****: We fully agree with the reviewer. There could be multiple reasons, including failure to honor triage referrals due to socio-economic challenges. This has now been added (line 344).*

**Comment 5**: In the manuscript it is not clear how the health providers delivered results. In fact, one of challenges reported was that the screening staff had difficulties in explaining results. Evidence have shown that HPV positivity can have an important negative impact on the psychosocial health of tested women. In addition, for many women, information provided by health providers about HPV is often confusing; women have difficulties in understanding HPV-test results and steps to follow. Both HPV’s psychosocial impact of HPV-testing and lack of information regarding the follow-up process might not only diminish women's quality of life but also reduce their retention to follow up. Other important point to explain the low follow up rate could be the wide range of TAT.

I suggest that authors may consider these points in Discussion section.

***Our Response****: we agree with the reviewer. Four possible explanations are now comprehensively captured; documentation, socio-economic challenges, cultural barriers and long TAT (lines 342-348).*

**Comment 6**: In line 376, remove dot: “placement of any new and innovative multiplex platforms that may come into the market in future.. A better”

***Our response****: we thank the reviewer for noticing this punctuation error. We have corrected it (line 396).*

**Second reviewer**

Integrating human papilloma virus testing as a point-of care service using GeneXpert platforms: 2 Findings and lessons from a Kenyan pilot study (2019-2020)

Some observations:

**Comment 1**: Abstract
Key Findings:
line 61: is 52.8% instead 53.8% please review

***Our response****: we thank the review for detecting the arithmetic error; it is actually 52.8%. It has now been corrected.*

**Comment 2**: Data management
Page 15: lines 201 and 202: table is not referenced in text.

***Our response****: we thank the reviewer for identifying this; it has now been corrected (line 214).*

**Comment 3: Findings**
Screening performance indicators Page 16:
Line 231: Table 1: HPV testing statistics, National HPV testing pilot project, Kenya, 2020: is not referenced in text.

***Our response****: this has now been corrected, as table 2 (line 239).*


**Comment 4**: Gene Xpert utilization
Page 19: Table 4: Change in utilization of gene Xpert equipment during the HPV pilot, Kenya, 2020: is not referenced in text.

***Response****: this has now been corrected, as table 5 (line 290).*

**Comment 5**: Page 19: Successes, challenges and opportunities
Table 5: Successes, challenges and opportunities identified during the nominal group discussions, Kenya HPV pilot study, 2020: is not referenced in text.

***Our response****: this has now been corrected, as table 6 (line 303).*

**Comment 6**: Line 277: is pandemic? Or panemic?

***Our response****: this was a typing error. It has now been corrected.*

**Comment 7**: Discussion

Page 23, line 364 is implementation

Conclusion and recommendations
Page 24 line 376: …. After future only a dot.

***Our response****: this has been corrected as well.*

We hope this adequately addresses the reviewers’ concerns and look forward to processing and final publication of this manuscript.

Sincerely,

Dr Valerian Mwenda,

Corresponding author

Email: [valmwenda@gmail.com](mailto:valmwenda@gmail.com), +254723992573
